# Supplementary material for: The Construction and Exploration of a Comprehensive MicroRNA Centered Regulatory Network in Foxtail Millet (Setaria italica L.)
Source: Front Plant Sci. 2022 May 6;13:848474. doi: 10.3389/fpls.2022.848474 (PMC9121102; doi:10.3389/fpls.2022.848474)
Supplement: Supplementary file 5 [file Data_Sheet_5.zip › Sit-miRNovel2_Seita.2G219400.1_1275_TPlot.pdf]

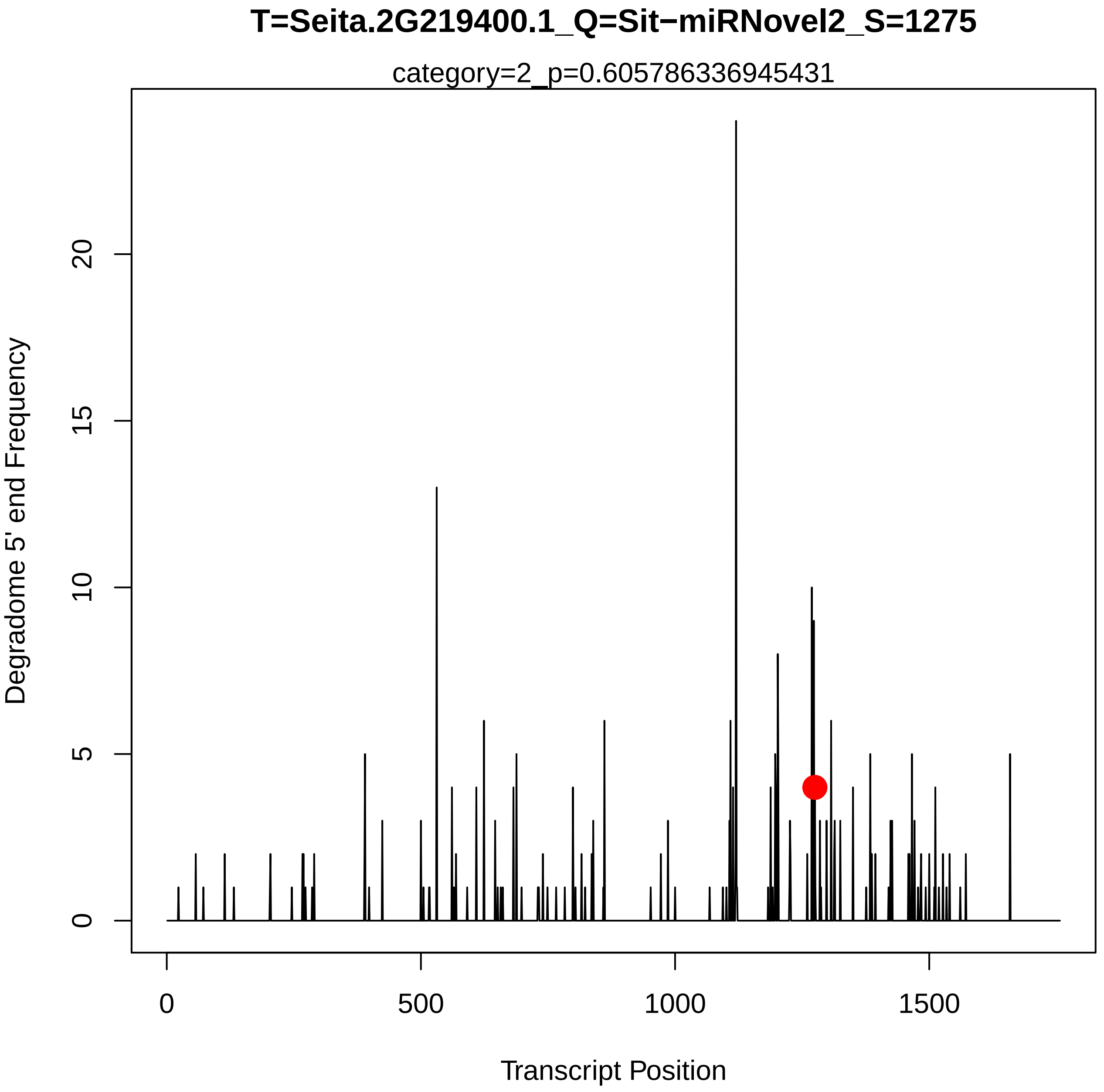
T=Seita.2G219400.1_Q=Sit−miRNovel2_S=1275
category=2_p=0.605786336945431
Degradome 5' end Frequency
0
5
10
15
20
0
500
1000
1500
Transcript Position
